# Promoting differentiation of cultured myoblasts using biomimetic surfaces that present alpha-laminin-2 peptides

**DOI:** 10.1007/s10616-016-0006-y

**Published:** 2016-08-09

**Authors:** Francine Parker, Kathryn White, Siȏn Phillips, Michelle Peckham

**Affiliations:** 1 School of Molecular and Cellular Biology, Faculty of Biological Sciences, The University of Leeds, Leeds, LS2 9JT UK; 2 Bioscience Centre, International Centre for Life, Orla Protein Technologies Ltd, Newcastle upon Tyne, NE1 4EP UK

**Keywords:** Myoblast, Myogenesis, Laminin, LAMA2, Peptides, Differentiation

## Abstract

Traditionally, muscle cell lines are cultured on glass coverslips and differentiated to investigate myoblast fusion and differentiation. Efficient differentiation of myoblasts produces a dense network of myotubes with the correct organisation for contraction. Here we have tested the ability of artificially generated, precisely controlled peptide surfaces to enhance the efficiency of myoblast differentiation. We focused on specific short peptides from α-laminin-2 (IKVSV, VQLRNGFPYFSY and GLLFYMARINHA) as well as residues 15–155 from FGF1. We tested if these peptides in isolation, and/or in combination promoted muscle differentiation in culture, by promoting fusion and/or by improving sarcomere organisation. The majority of these peptides promoted fusion and differentiation in two different mouse myogenic cell lines and in primary human myoblasts. The additive effects of all four peptides gave the best results for both mouse cell lines tested, while primary human cell cultures differentiated equally well on most peptide surfaces tested. These data show that a mixture of short biomimetic peptides can reliably promote differentiation in mouse and human myoblasts.

## Introduction

Muscle cell lines, originally derived from satellite cells are commonly used to study muscle fusion and differentiation in tissue culture. However, levels of fusion and differentiation can be variable depending on the method of culture and the cell lines used. Our challenge was to develop a more reliable method to generate well-differentiated myotubes in culture for this purpose. In vivo, new muscle fibres develop within a complex thin layer of extracellular matrix found adjacent to the plasma membrane (sarcolemma) of muscle fibres, known as the basal lamina. We therefore wanted to test if specific peptides derived from key components of the basal lamina would promote myoblast survival and differentiation in culture.

A major constituent of the basal lamina of adult skeletal muscle is Laminin 211 (also known as merosin). Laminin is a heterotrimeric extracellular glycoprotein, consisting of an α, a β and a γ chain (Ehrig et al. 1990). We have previously shown that myoblast survival and fusion into myotubes depends on laminin 111 (which is made up of α1, β1 and γ1 chains), and not on fibronectin or vitronectin (Clark et al. 1997). However, the major constituent of the basal lamina of adult skeletal muscle is not laminin 111, but laminin 211 (Sanes 2003), in which the α chain is LAMA2 (α2). Laminin 211 has also been reported to be important for myoblast fusion and myotube survival (Vachon et al. 1996).

Laminin 211 plays a key role in promoting muscle cell adhesion to the extracellular matrix and thus in cell survival. It binds to the membrane proteins α-dystroglycan and integrin α7. α-dystroglycan is part of the dystrophin associated glycoprotein complex, which is associated with the actin cytoskeleton within the cells. Through binding to laminin 211, it thus connects the extracellular matrix through to cytoskeletal γ actin on the inside of the cell. α7β1 is the main integrin expressed in adult skeletal muscle (Song et al. 1992). It exclusively binds to laminin and is upregulated on myoblast fusion (Song et al. 1992; Yao et al. 1996). This latter interaction promotes several cell survival pathways (reviewed in Holmberg and Durbeej 2013). Mutations in laminin 211 cause muscular dystrophy in mice and humans (Tome et al. 1994; Xu et al. 1994a, b). The commonly used muscle cell line, C2C12 (Blau et al. 1983) expresses LAMA2 both as myoblasts and myotubes (Schuler and Sorokin 1995), while α-dystroglycan is expressed at lower levels in myoblasts compared to myotubes (Kostrominova and Tanzer 1995). The presence of α-dystroglycan in myoblasts is known to promote myoblast adhesion (Thompson et al. 2010).

Mapping studies have shown that the α-chain of laminin 211 (LAMA2) binds to α-dystroglycan through its lamin G (LG) domains: LG1-3 and LG4-5 (Talts et al. 1999). A more recent detailed peptide mapping study showed that two peptides within LG4, with the amino acid sequences of VQLRNGFPYFSY and GLLFMARINHA strongly and specifically bound α-dystroglycan, while GLLFMARINHA also bound to heparin (Suzuki et al. 2010). The arginine residues in both of these peptide sequences have been identified as important in binding to α-dystroglycan (Suzuki et al. 2010; Wizemann et al. 2003). A synthetic peptide with the sequence IKVAV (Ile-Lys-Val-Ala-Val), which is found just upstream of the first LG domain from laminin α1 is also known to be important in cell adhesion (Tashiro et al. 1989) and to interact with integrins (Freitas et al. 2007). The equivalent sequence in laminin α2 is the conserved IKVSV.

A variety of other bioactive polypeptides are also important in myogenesis. These include fibroblast growth factors (FGFs), which are bound to proteins in the matrix in vivo. For example, syndecan-3 and -4, which are transmembrane heparan sulphate proteoglycans expressed by muscle satellite cells, regulate FGF signaling in proliferating satellite cells. This appears to be required for activation and proliferation of satellite cells (Cornelison et al. 2001). While high levels of FGF1 may inhibit myogenesis, FGF1 can promote differentiation and exogenous FGF1 can also prevent apoptosis in neuronal cells (Desire et al. 1998). Traditionally, growth factors such as FGF1 are added to cell culture media, and there is increasing evidence these growth factors remain biologically active when immobilized onto surfaces (Feito et al. 2011). Therefore FGF1 may also help promote myogenesis of cultured myoblasts.

Recently, a novel approach has been implemented to display peptides known to be important for cell adhesion by Orla Protein Technologies (Cooke et al. 2008). Peptides are engineered into the extracellular loops of the bacterial outer membrane protein A (OmpA). The purified protein is then applied stereo-specifically onto a gold-coated surface via a cysteine residue in OmpA, such that an oriented monolayer forms, with peptides all facing upwards, accessible to the cells in a functional conformation (Cooke et al. 2008) and at a concentration that is precisely controlled. Gaps between the protein molecules are filled in using thiolipids or thioalkanes, which means that only the peptide motifs being tested are presented to the cells. A single peptide or a mixture of different peptides can also be used. This approach allows precise control over concentration and make-up of ECM peptide motifs presented to the cells.

**Fig. 1:**
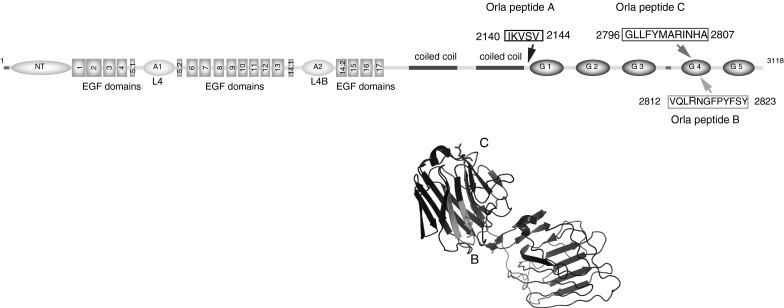
Diagrammatic representation of laminin α2, and the location of the peptides used. The positions of peptides B and C are additionally shown on the structure for the G4 and G5 domains from laminin α2 (PDB:1DYK). *Highlighted* ‘*R*’ in Orla peptides B and C may be critical for binding of α-dystroglycan (Wizemann et al. 2003)

Here, we have used this approach to test the abilities of the three LAMA2 peptides described above (Fig. 1; Table 1), together with an FGF1 peptide in promoting myoblast fusion and differentiation. The FGF peptide consisted of amino acids 15–155 of the human FGF1, which was engineered into the N-terminus of OmpA with a 16 amino acid linker sequence to enhance the presentation of FGF1. We tested if any of these peptides, alone, or in combination, promoted fusion and/or differentiation in culture, compared to the traditionally used glass coverslip, coated with gelatin. We further tested if a non-specific peptide (c-myc) was able to support fusion and differentiation, as a negative control for our experiments.

**Table 1:** Orla surface key: sequences of the peptides used in these experiments

Orla	Sequence
A	IKVSV (2138–2142 in LAMA1)
B	VQLRNGFPYFSY (residues 2806–2817 in LAMA1)
C	GLLFYMARINHA (residues 2790–2801 in LAMA1)
D	FGF1 (residues 15–155)
A + D	IKVSV & FGF1 (50:50)
B + D	VQLRNGFPYFSY & FGF1 (50:50)
C + D	GLLFYMARINHA & FGF1 (50:50)
A + B + C	IKVSV & VQLRNGFPYFSY & GLLFYMARINHA (33:33:33)
A + B + C + D	IKVSV & VQLRNGFPYFSY & GLLFYMARINHA & FGF1 (25:25:25:25)
c-Myc	NNKLISEEDL

Our overall aim was to develop a reliable, consistent method of differentiating cultured muscle cells, such that level of fusion of myoblasts into myotubes and myofibrillogenesis (the production of myofibrils, into which muscle contractile proteins are precisely organised) is high. Currently, differentiation of cultured myoblasts, particularly using standard muscle cell lines such as the C2C12 cells, does not always give reliable results. In particular, while the myoblasts might fuse into multinucleated myotubes, the level of sarcomere organisation within these myotubes can be very low. This makes it challenging to use these cells to investigate muscle differentiation, and sarcomeric organisation. We tested three different types of cultured muscle cells: the commonly used mouse C2C12 cell line (Blau et al. 1983), a conditionally immortalised mouse muscle cell clone, C1F, developed by us from the ‘immorto’ mouse (Morgan et al. 1994; Peltzer et al. 2008), and primary human skeletal muscle cells that are commercially available.

## Materials and methods

### Skeletal muscle cell culture

C2C12 myoblasts [purchased from PHE (Public Health England) culture collections: ECACC, Porton Down, Salisbury, UK] were cultured in growth media: DMEM (Dulbecco’s Minimum Essential Medium, with high glucose, and containing Glutamax) (Gibco, Grand Island, NY, USA) with 20 % FCS (Fetal Calf Serum) (GIBCO) and 1 % penicillin/streptomycin (GIBCO) following the recommended protocol, and differentiated in DMEM supplemented with 2 % equine serum and 1 % penicillin/streptomycin. C1F cells were isolated as a clonal line from neonatal muscle, from the H2 Kb-tsA58 transgenic mouse (Morgan et al. 1994) in the Peckham laboratory (Peltzer et al. 2008), and cultured as described previously (Morgan et al. 1994; Peltzer et al. 2008). Briefly, myoblasts were proliferated at 33 °C in the presence of IFN-γ (Becton Dickinson Biosciences, San José, CA, USA) in standard growth medium (DMEM, high glucose, Glutamax, 20 % FCS, 1 % chick embryo extract (CEE) (CEE was prepared as described by Dabiri et al. 1999. Briefly, 120 embryos from 11-day-old fertilised eggs (Henry Stewart and Co.) were extracted from the yolk sac and washed in PBS before being decapitated and homogenised through a 20 ml syringe. An equal volume of DMEM (Gibco) is then added and the mixture left to sit at room temperature for 1.5 hours, with occasional swirling, before being aliquoted and stored at −20 °C), 1 % penicillin/streptomycin) and differentiated into myotubes at 37 °C in the absence of IFN-γ in differentiation medium (DMEM, 5 % FCS, 2 % CEE, 1 % penicillin/streptomycin). Primary human skeletal muscle cells were purchased from Public Health England [PHE: HSkMC (150-05F)], and cultured and differentiated as recommended in growth and differentiation medium additionally purchased from PHE. Cells were allowed to differentiate for 5 days.

### Orla surfaces

Orla (Orla Peptides were provided by Orla Protein Technologies Ltd., Bioscience Centre, Newcastle Upon Tyne, UK) surfaces were produced using the previously described method (Cooke et al. 2008). Briefly, cleaned glass coverslips, coated with a 25 nm layer of gold were cleaned and used to attach the Orla peptides via a cystein residue. The coverslips were coated in protein using 3 × 1 h incubation steps, using 250 μL protein solution for each incubation, with the Orla peptide at a concentration of 0.1 μM. This results in a concentration of the Orla peptides at approximately 150 ng/cm^2^. Where surfaces were coated with more than one protein, the proteins were mixed at equal molarity and applied to surface as a mixed protein solution. For example, when a mixture of 4 peptides was used, they were mixed in equal parts (25 % for each peptide).

A variety of Orla surfaces (Table 1) were used with either contained single peptides (for each peptide tested) or a mixture of peptides. 3 LAMA2, and an FGF1 peptide were tested (Table 1). In addition, control c-myc peptides were tested for C2C12 and C1F cells. Gelatin (0.1 % w/v) coated glass (Porcine skin gelatin (Sigma) at 0.1 % concentration in water, filter sterilized; a small drop was added to the coverslip and left for 1–2 mins, then aspirated off and allowed to air dry in the tissue culture hood, before adding the cells), which is commonly used to coat glass for cultured muscle cell differentiation, was used as a additional control for all cell lines tested. The Orla surfaces were sterilised with a 15 min incubation in 70 % ethanol before cell seeding. The Orla peptides are covalently linked to the glass surfaces, via silane thiol chemistry, making removal of the peptides by the cells during cell culture unlikely.

In total, we performed 3 experiments for C2C12 cells. To compare these results with those for other cell lines, we additionally performed 2 separate experiments for C1F cells. We only performed 1 experiment for primary HuSK cells as these cells can only be passaged for a limited amount of time. All of the surfaces listed (Table 1) were tested in each experiment as well as controls using gelatin coated glass coverslips only.

### Proliferation assays

Proliferation assays were performed for a subset of the Orla surfaces, using C2C12 and C1F myoblasts only. C2C12 and C1F myoblasts were plated onto the Orla surfaces at a density of 1 × 10^4^ per mL. Images were taken using a CytoMate camera (Cytomate Technologies, B.V., Eindhoven, The Netherlands) in which the cell culture dishes are placed on the cytomate, and images taken of the cells as they grow while in the cell culture incubator, minimising any disturbances to the cells. A 10× objective was used. Images were taken 2 h after plating (t = 0 h for analysis) and every 24 h until cells reached confluency (>90 %). The cell coverage function in the Cytomate software was used to measure cell proliferation. 3 images were taken at each time point and the experiment was performed in triplicate.

### Immunostaining

To perform immunostaining, first the cells were fixed with freshly made 2 % paraformaldehyde (PFA) in phosphate buffered saline (PBS) for 15–20 min. Cells were then permeabilised with 0.5 % Triton-X in PBS and incubated with murine primary antibodies that recognise all isoforms of skeletal myosin (A4.1025). The antibodies were obtained from hybridoma cultures in the Peckham laboratory, using hybridomas originally derived by Helen Blau (Maggs et al. 2000). The secondary antibody used was anti-mouse Alexa Fluor 488 (Invitrogen Molecular Probes, Eugene, OR, USA) and DAPI (Molecular Probes) was used for nuclear visualisation.

Images of fixed and stained cells were obtained using a Deltavision deconvolution system at either low magnification (using the 20× objective) to analyse fusion, or at high magnification (using the 100× objective) to analyse sarcomere organisation. Images taken at the higher magnification involved taking a Z-stack of images, followed by a deconvolution process, and then the entire Z-stack was combined into a final projection image prior to analysis. Additional tiled images were obtained using the Zeiss LSM 880 microscope (Oberkochen, Germany), using the 40× N.A. 1.4 oil objective to image a large field of view at high magnification to further compare fusion in the different cell lines.

### Analysis of fusion and sarcomere organisation

Low magnification images of differentiated cells (as described above), that were fixed and stained for skeletal myosin and nuclei (using DAPI) were used to calculate the fusion index for the differentiated cells after 5 days of differentiation. The total number of nuclei in each field of view was counted together with the total number of nuclei within myotubes (which were stained positively for myosin heavy chain). This analysis was repeated for a minimum of 5 fields of view per surface, per experiment. The fusion index was then calculated as the proportion of nuclei within myotubes as a function of the total number of nuclei. This method of estimating fusion is a commonly used approach in the muscle field. Fusion indices were calculated separately by 3 researchers and the data combined.

Sarcomeric organisation of the high magnification projection images was analysed using ImageJ. Lines, with a fixed length, were drawn randomly within each image, and the line intensity profile along the lines was measured. A minimum of 3 Z-stack maximum projection images were used for each ORLA surface, and 4 lines were drawn per image; resulting in a minimum of 12 graphs per surface, per experiment. Line profiles for myotubes in which sarcomeric organisation is highly organised generated graphs with defined peaks and troughs, while line profiles for myotubes in which sarcomeric organisation was low, lacked regular features (examples are shown in Fig. 5b). The number of peaks per graph with the correct periodicity for sarcomeric banding (around 2 μm) were measured and averaged per surface. As with fusion indices, these measurements were performed by three separate researchers, and the data combined.

## Results and discussion

### Fusion and proliferation

**Fig. 2:**
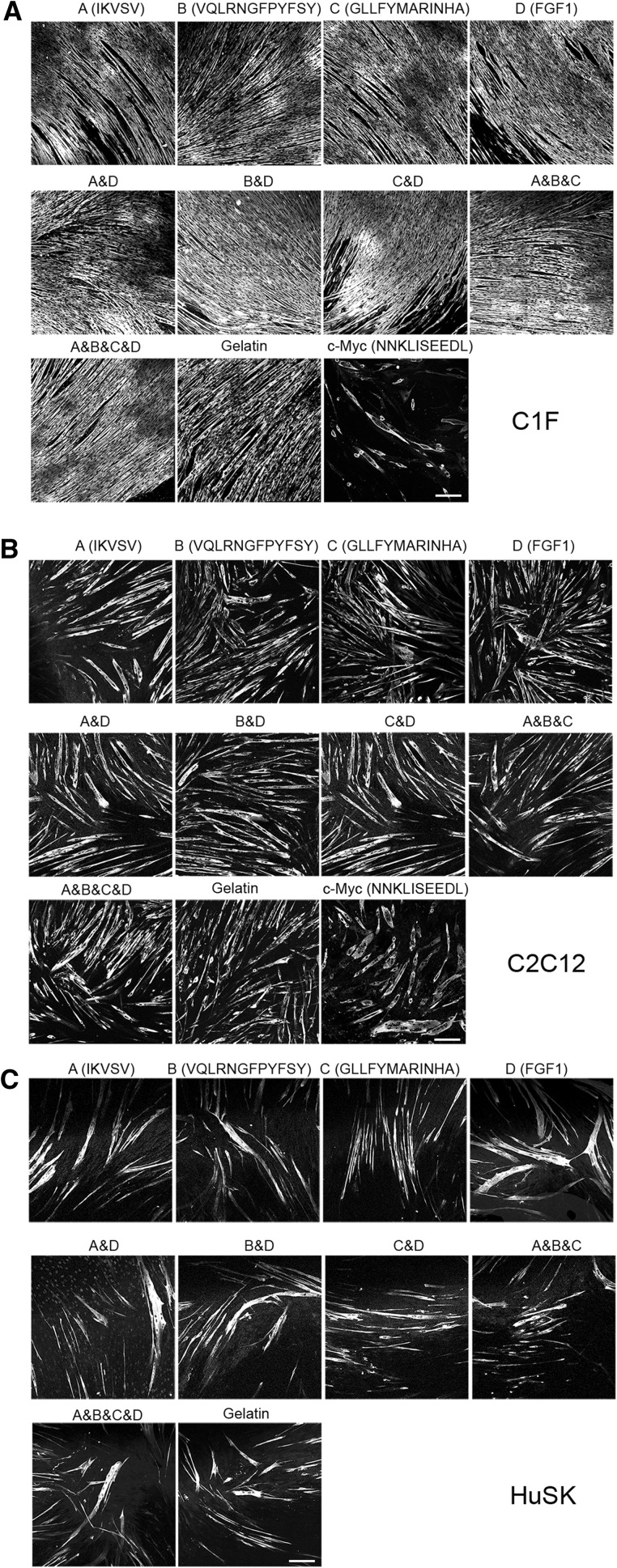
Effects of different surfaces on fusion for mouse and human cells. The images shown are of 5 day myotubes, fixed and stained for skeletal myosin, which is only expressed in differentiated cells, the majority of which are multinucleated myotubes. Results for the two mouse muscle cell lines, with C1F are shown in **a** and C2C12 in **b** and these results include a further test on an Orla surface in which the peptide presented is c-Myc, which is not expected to promote myoblast fusion and differentiation. For comparison, results for primary human skeletal muscle cells (HuSK) are shown in **c**. These cells were not tested for differentiation on the c-Myc surface. *Scale bar* is 200 μm

The ability of the cultured myoblasts to fuse strongly depended on cell type (Fig. 2). C1F myoblasts showed the greatest ability to fuse, while human skeletal myoblasts (HuSK) and C2C12 myoblasts were less efficient at fusing. The ability of the myoblasts to fuse is demonstrated by confocal images of the myotubes formed following fusion of the myoblasts on the different surfaces, after 5 days under differentiation conditions (Fig. 2). These cells have been fixed and stained for skeletal muscle myosin, which is only expressed once the cells have differentiated. For the mouse myoblast line C1F, the surfaces are almost completely covered by long, skeletal myosin positive, myotubes. For the mouse myoblast line C2C12, while myotubes are present, there are fewer of them, although all the cells were plated at the same density in each experiment. HuSK myoblasts were similar to C2C12 myoblasts in terms of fusion.

**Fig. 3:**
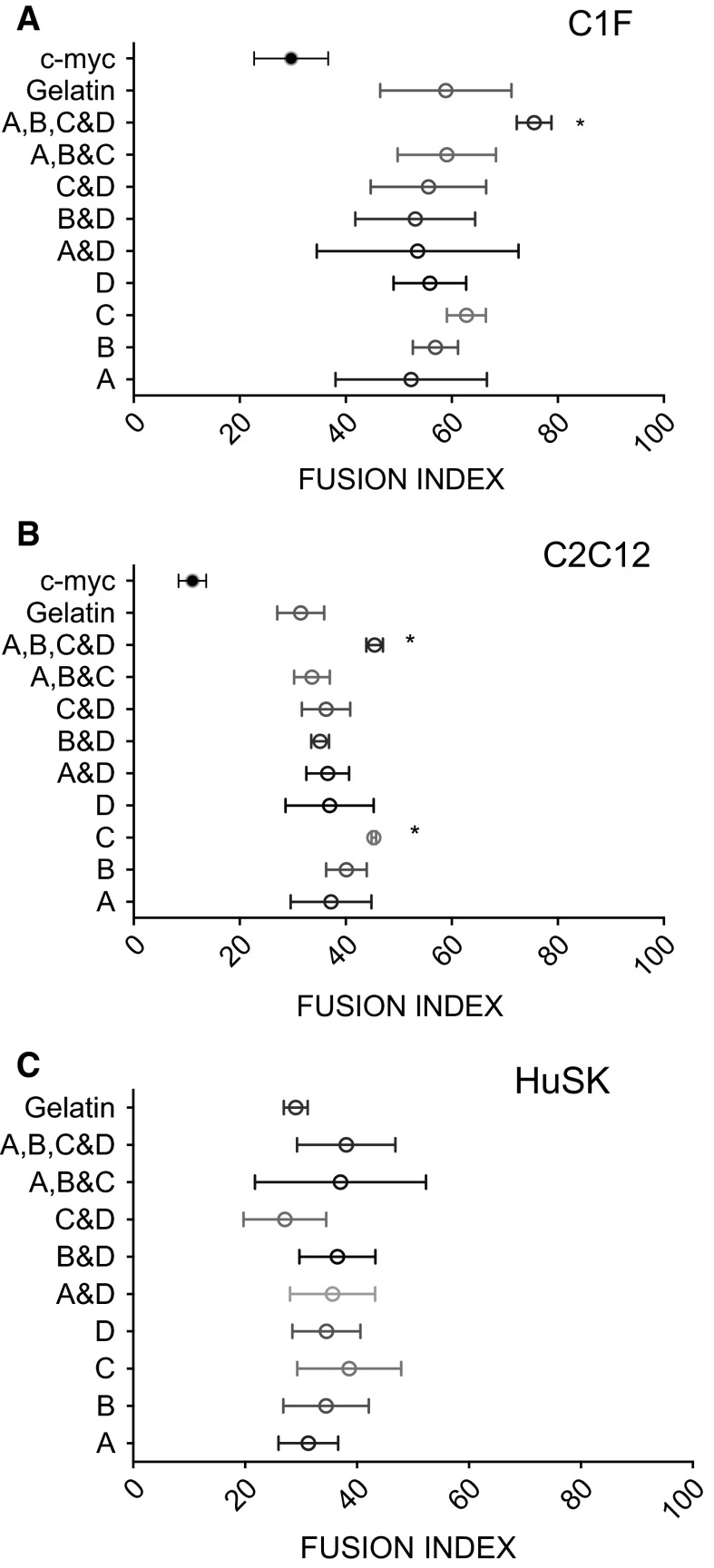
Analysis of fusion for C1F, C2C12 and HuSk cells. Data show the mean ± SD for three experiments (C2C12 in **b**), and two experiments (C1F in **a**). For HuSk (**c**), the data shown are the mean ± SD for the individual measurements of fusion from 5 different regions, from a single experiment. *Significant difference from cells cultured on gelatin (*p* < 0.05). Almost all the surfaces showed a significant increase in fusion compared to fusion on c-myc Orla peptides for C2C12 and C1F (HuSK was not tested for c-myc), apart from A&D and A (for C1F)

Quantification of fusion, which measures the numbers of nuclei present in a field of view, and how many of these nuclei are present in the myotubes, showed that C1F cells had the highest fusion index (Fig. 3), in that the majority of the nuclei were in multinucleated myotubes (~60–80 % on most surfaces). The fusion index obtained for human skeletal muscle cells (HuSK) and C2C12 cells was similar, and lower than that obtained for C1F cells at ~40 % on average (Fig. 3).

Tests of the single LAMA2 peptides for all three cell types, showed that Orla C (with the sequence GLLFYMARINHA, Table 1) was the best in promoting fusion for all three cell types, as quantified by the fusion indices obtained. While the differences in the fusion indices for the different single peptides were generally small, fusion on OrlaC was significantly higher for C2C12 myoblasts than fusion on gelatin, which is commonly used as a substrate for fusion and differentiation of myoblasts. In contrast, fusion of C1F and C2C12 cells on the c-myc Orla peptide, which is not expected to support fusion, was significantly reduced compared to fusion on gelatin, and to fusion on Orla C, for C2C12 and C1F cells. Thus, the c-myc peptide is unable to support fusion, while the LAMA2 peptides promote fusion for all the cell lines tested.

Tests for the mixture of pepides showed that an equimolar mixture of all three LAMA2 peptides, together with FGF1 (Figs. 2, 3) increased fusion significantly for both C2C12 and C1F cells, compared to differentiation on a gelatin only surface, or on c-myc peptide surfaces. In contrast, mixtures of FGF1 and one of the LAMA2 peptides (A&D, B&D or C&D) did not improve fusion compared to the single LAMA2 peptides alone. In addition, the mixture of all three LAMA2 peptides (A&B&C) was not markedly different from the results obtained for each peptide in isolation. A similar trend was observed for HuSK cells, although this increase in fusion was not significant. These results suggest that each peptide is reasonably good in promoting fusion in isolation, and they do not work synergistically, unless all 3 LAMA2 peptides and the FGF1 peptide is present. This effect was most marked for the two mouse muscle cell lines, C1F and C2C12.

**Fig. 4:**
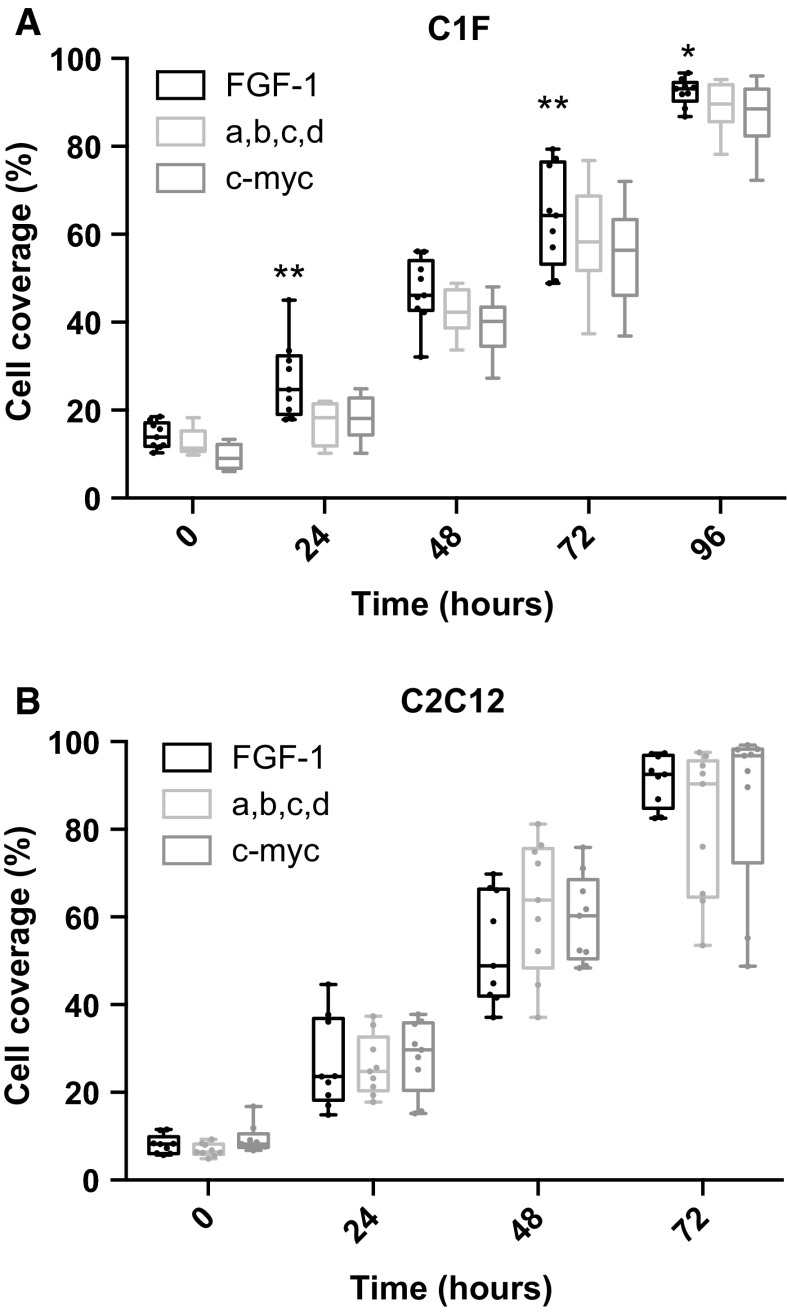
Analysis of cell proliferation for C1F and C2C12 cells on selected surfaces. The rate of increase of cell coverage on FGF-1, the mixture of all 4 peptides (A&B&C&D: FGF-1, and the three laminin peptides), as well as c-myc was measured over 96 h. Experiments were conducted in triplicate. Statistical differences are shown as *p < 0.05; **p < 0.001

To test if this effect on fusion was due to differences in proliferation of the cells prior to fusion, we analysed rates of proliferation on three of the surfaces: FGF1 alone, the mixture of all three LAMA2 peptides together with FGF1 (A&B&C&D), and the c-myc peptide (Fig. 4). None of these surfaces affected proliferation of the C2C12 myoblasts, confirming that any differences in fusion observed were not due to changes in proliferation. For the C1F myoblasts, FGF1 did increase proliferation significantly above that observed for the mixture of all 4 peptides (A&B&C&D), and c-myc. However, the rate of proliferation for C1F cells on the c-myc peptide, and the mixture of all 4 peptides was not significantly different. As fusion on the mixture of all 4 peptides was significantly higher than in the other two conditions, we can conclude any effects on proliferation are not the underlying cause of improvements in fusion.

### Sarcomere organisation

**Fig. 5:**
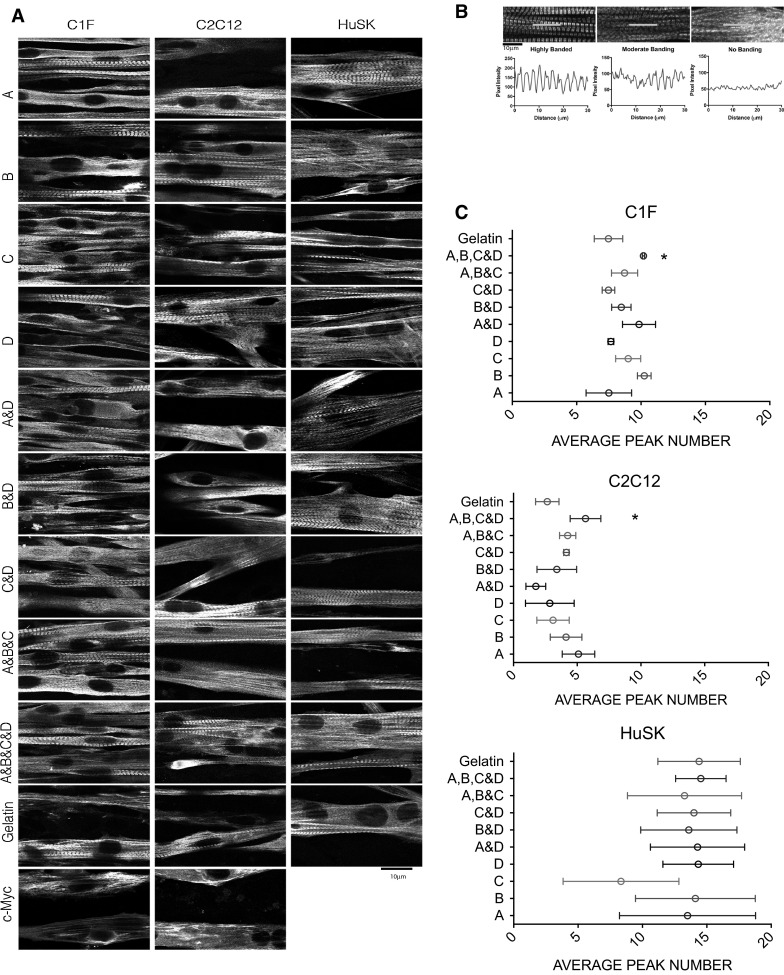
Differentiation of mouse and human myogenic cell lines on different surfaces. **a** Images of myotubes for each type of surface, for C1F (mouse), C2C12 (mouse) and HuSK (human) cells. (C-myc was not tested for HuSK cells). **b** Analysis of sarcomere organisation (see “Materials and methods”). Sarcomeres can range from highly organised (multiple peaks/highly banded) to medium to low (no banding). The intensity profiles along the *red lines* shown in these images were used to generate the intensity profiles shown below. **c** Analysis of sarcomere organisation for C2C12 (mouse cell line), C1F (mouse cell line;) and human skeletal muscle cells. Values shown are the mean ± SD values from two experiments for C1F and C2C12, and 1 experiment for human skeletal muscle cells (showing the mean ± SD, for n = 15–18 observations). *Significant difference from cells cultured on gelatin (*p* < 0.05). Values for results on nc-myc are not shown in this analysis, as cells with a sarcomeric arrangement were only rarely found (see **a**). (Color figure online)

Sarcomere organisation also varied markedly between the three different cell types used (Fig. 5). Sarcomere organisation was best for the differentiated HuSK myotubes, intermediate for C1F myotubes, and worst for C2C12 myotubes (Fig. 5c). This was quantified by measuring the number of peaks along a line of fixed length across multiple myotubes in multiple fields (see methods). The higher the average number of peaks, the more likely that the majority of myotubes contain a more highly striated organisation of skeletal myosin, and thus good sarcomere organisation. These results show that sarcomere organisation does not necessarily correlate with the ability of the cells to fuse, as HuSK myoblasts had a similar fusion index to C2C12 cells, but showed better sarcomere organisation, whereas C1F cells fused well, but had intermediate levels of sarcomere organisation.

Tests of the Orla peptides showed that, as with fusion, the best sarcomere organisation for C1F cells was obtained for the mixture of all 4 peptides (Fig. 5) and the improvement in sarcomere organisation was significantly increased compared to that obtained on gelatin. This suggests that surfaces that promote fusion for individual cell types, can also support better levels of sarcomere organisation, as C1F cells fused best on the mixture of all 4 peptides, and sarcomere organisation was also best on that surface. However, for single peptides, Orla B (GLLFYMARINHA), rather than C showed the best results for sarcomere organisation. Adding in FGF1 to single LAMA2 peptides slightly improved results for IKVSV (A&D), but not the other two LAMA2 peptides. Generally, sarcomere organisation was improved on all of the Orla surfaces, compared to gelatin alone. C-myc peptides were very poor at promoting sarcomere organisation, and almost no myotubes were observed with recognisable banded structures (Fig. 5a).

For C2C12 cells, the best sarcomere organisation was again obtained for the mixture of all 4 peptides (Fig. 5), again linking an improvement in fusion with that of differentiation for an individual cell type. The improvement in sarcomere organisation was significantly increased compared to gelatin. The best single peptide was IKVSV (A). Adding in FGF1 to the single LAMA2 peptides did not improve sarcomere organisation beyond that of the LAMA2 peptides alone. In general, all the peptides improved sarcomere organisation compared to gelatin alone. As with CIF cells, C-myc peptides were very poor at promoting sarcomere organisation, and almost no myotubes were observed with recognisable banded structures (Fig. 5a).

HuSK cells appeared to have well organised sarcomeres on most surfaces, with the exception of Orla C. As their sarcomeric organisation is already the highest compared to the other cell lines, it is possible that Orla peptides have less effect on their sarcomere organisation compared to C1F and C2C12 cells.

These data show that the extent to which each of the different types of cultured cells (C1F, C2C12 and HuSk) can fuse and differentiate is largely determined by the inherent properties of these cells. However, use of the Orla surfaces is able to improve fusion and differentiation above that of using gelatin, and is significantly better than using an unrelated peptide such as c-myc, which is not expected to promote fusion. Moreover, using the combination of all 3 LAMA2 peptides and FGF1 significantly improved fusion and differentiation for the two mouse myogenic cell lines (C1F and C2C12), and was the best surface for the human myogenic (HuSK) cells. It is also noticeable that there is less variation in the fusion index and sarcomere organisation on this particular surface compared to the other surfaces used. For fusion, GLLFYMARINHA appears to be the most effective of the three LAMA2 peptides we tested, when used as a single peptide.

Overall, the combination of all three of the LAMA2 peptides with FGF1 was best for both fusion and differentiation for the two mouse myogenic cell lines (C2C12 and C1F). This combination was also better for HuSK cells. It is interesting that fusion and differentiation can be improved by adding FGF1 to the mixture of all three LAMA2 peptides, although the combination of FGF1 with any of the LAMA2 peptides in isolation is less effective. Exogenous FGF1 might be expected to promote proliferation rather than differentiation (Uruno et al. 1999). However, using the mixture of all 4 peptides did not show significant effects on proliferation for C2C12 cells, and only a small increase for C1F cells, although this was significant. Although the FGF1 is covalently coupled to the glass surface via the Orla peptide, other studies have shown that these growth factors remain biologically active when immobilized onto surfaces (Feito et al. 2011). Thus, we suspect that when used in combination with the three LAMA2 peptides, FGF1 is able to promote myotube survival, and the slightly lower relative concentration of FGF1 in the mixture of all 4 peptides, where FGF is at 25 % of the concentration compared to a surface that only contains the FGF1 peptide, is better optimised for promotion of proliferation and myoblast survival prior to and during differentiation, than the higher levels of FGF1 present in the mixtures with a single peptide. Alternatively, there is a synergistic effect between FGF1 and the 3 LAMA2 peptides that promotes myotube differentiation.

In conclusion, using the three LAMA2 peptides in combination with FGF1 promotes fusion and differentiation, and gives more consistent results for each of the cell lines tested here, and should be useful for studies that require well-differentiated cultured muscle cells in the future.
